# Independent impacts of age and hearing loss on spatial release in a complex auditory environment

**DOI:** 10.3389/fnins.2013.00252

**Published:** 2013-12-23

**Authors:** Frederick J. Gallun, Anna C. Diedesch, Sean D. Kampel, Kasey M. Jakien

**Affiliations:** ^1^Department of Veterans Affairs, National Center for Rehabilitative Auditory Research, Portland VA Medical CenterPortland, OR, USA; ^2^Otolaryngology/Head and Neck Surgery, Oregon Health and Science UniversityPortland, OR, USA; ^3^Hearing and Speech Sciences, Vanderbilt UniversityNashville, TN, USA

**Keywords:** spatial release, aging, hearing loss, virtual spatial array

## Abstract

Listeners in complex auditory environments can benefit from the ability to use a variety of spatial and spectrotemporal cues for sound source segregation. Probing these abilities is an essential part of gaining a more complete understanding of why listeners differ in navigating the auditory environment. Two fundamental processes that can impact the auditory systems of individual listeners are aging and hearing loss. One difficulty with uncovering the independent effects of age and hearing loss on spatial release is the commonly observed phenomenon of age-related hearing loss. In order to reveal the effects of aging on spatial hearing, it is essential to develop testing methods that reduce the influence of hearing loss on the outcomes. The statistical power needed for such testing generally requires a larger number of participants than can easily be tested using traditional behavioral methods. This work describes the development and validation of a rapid method by which listeners can be categorized in terms of their ability to use spatial and spectrotemporal cues to separate competing speech streams. Results show that when age and audibility are not covarying, age alone can be shown to substantially reduce spatial release from masking. These data support the hypothesis that aging, independent of an individual's hearing threshold, can result in changes in the cortical and/or subcortical structures essential for spatial hearing.

## Introduction

Because a listener relies on multiple spatial and spectrotemporal cues to accurately segregate sound sources, there are many neural processes that support auditory scene analysis. Two of the most important acoustical cues, differences in the pitches and spatial locations of the sound sources, depend on accurate neural timing for these cues to be completely encoded by the central auditory system (Moore, 2007). However, reductions in the accuracy of neural timing may have little impact on an individual's ability to detect energy at a particular frequency. This may explain why an audiogram conducted in quiet over headphones does not correlate well with an individual's ability to use temporal cues (Durlach et al., 1981; Buus et al., 1984; Smoski and Trahiotis, 1986; Gabriel et al., 1992; Koehnke et al., 1995; Lacher-Fougere and Demany, 2005; Ross et al., 2007; Strelcyk and Dau, 2009; Grose and Mamo, 2010; Ruggles et al., 2011). An additional issue that makes prediction of suprathreshold hearing abilities difficult is the presence of age-related hearing loss in many of the older participants. While it is possible that hearing loss and aging both reduce neural timing, animal studies (e.g., Caspary et al., 1995, 2008; Helfert et al., 1999; Wang et al., 2009; Scheidt et al., 2010; Walton, 2010) suggest that the two may act at different levels of the auditory system. For example, the impacts of hearing loss may be more related to reductions in the response of the auditory nerve, while the impacts of aging may be more related to impaired temporal coding in the auditory brainstem. This implies that it is very important to develop methods by which the impacts of aging can be revealed even in the presence of hearing loss.

One of the reasons for limited evidence in humans of the distinction between the neural processes associated with aging and those associated with hearing loss (beyond the limited ability of researchers to make direct measurements of the auditory nerve and brainstem in humans) is that the age of the listener has often been allowed to covary with hearing loss. Studies that have controlled for age effects (e.g., Hawkins and Wightman, 1980; Dubno et al., 2002, 2008) have found that spatial hearing can be substantially reduced in listeners with hearing loss, but to date no studies have effectively controlled for hearing loss, primarily due to issues of sample size.

One of the key abilities associated with binaural processing is spatial release from masking, which occurs when the ability to detect or identify a target sound (often speech) in the presence of one or more masking sounds is improved by spatial differences between target and masker(s) (Carhart et al., 1969; Hawley et al., 2004; Best et al., 2005; Gallun et al., 2005; Brungart and Simpson, 2007). While it has been fairly well established than the benefit obtained by hearing-impaired listeners in a multitalker segregation task is less than that obtained by normally-hearing listeners (Gelfand et al., 1988; Arbogast et al., 2005; Best et al., 2010; Hopkins and Moore, 2010), the impacts of age on spatial release from masking are less understood. In one of the best controlled studies with the largest sample of subjects, Marrone et al. (2008) were unable to clearly distinguish the effects of age from the effects of hearing loss due to a lack of statistical power. The experiments described here were designed to overcome the statistical lack of power common in previous reports in order to provide the strongest test possible of the hypothesis that aging, independent of hearing loss, can result in significant reductions in spatial release from masking.

## Experiment one: adaptive tracks; anechoic chamber

The goal of the first experiment was to document the ways in which the ages and hearing levels of the participants interacted with the spatial and pitch cues available in the stimuli to produce a particular level of speech identification performance.

### Materials and methods

Spatial release from masking was tested using stimuli drawn from the Coordinate Response Measure (CRM; Bolia et al., 2000), which consists of a simple corpus of sentences with the form “Ready (CALL SIGN) go to (COLOR) (NUMBER) now.” There are eight possible call signs (Arrow, Baron, Charlie, Eagle, Hopper, Laker, Ringo, Tiger), and 12 keywords: four colors (red, green, white, and blue) and the numbers 1–8. All possible combinations of the call signs, colors, and numbers are spoken by four male and four female talkers. The CRM corpus consists of high-quality recordings of each of the eight talkers saying all 256 possible combinations of call signs and keywords. The intelligibility of each of the keywords as spoken by each of the talkers was examined by Brungart (2001), who showed that there is no significant advantage to the listener of being asked to identify any one of the potential keywords.

Each listener was presented with a set of three simultaneous utterances from the CRM corpus and the goal was to attend to one of the sentences, identified by the callsign “Charlie.” Each sentence was presented from one of seven loudspeakers arranged at 15° separation in front of the listener in an anechoic chamber containing a ring of 24 loudspeakers surrounding the listener, each at approximately head height and at a distance of 1 m from the listener's head. Four spatial configurations were used: colocated (all three sentences presented from 0° azimuth), 15° separation (target at 0°, one masker at + 15° and another masker at −15°), 30° separation (target at 0°, maskers at ±30°), and 45° separation (target at 0°, maskers at ±45°). In addition, each spatial condition was tested in four talker-gender combination conditions: male/male (male target, male maskers), male/female (male target, female maskers), female/female (female target, female maskers), and female/male (female target, male maskers).

Identification thresholds, in terms of target-to-masker ratio (TMR), were estimated based on the average of four adaptive tracks for each of the 16 combinations of four spatial and four talker-gender conditions. In the masked conditions the target level was fixed at 50 dB SPL and the masker levels were adaptively varied, using a one-up/one-down procedure (estimating 50% correct; Levitt, 1971). Masker sentences each started at a level of 40 dB and were changed in level by 5 dB until three reversals were obtained, at which point the step size was changed to 1 dB and six more reversals were measured. The threshold was estimated to be the average of the last six reversals. TMR is expressed as the difference in level between the target and each masker. Thus, if the target level is 50 dB SPL and each masker is also 50 dB SPL, the TMR is 0 dB^1^.

To ensure audibility of the target sentence and familiarize listeners with the testing procedure, the first test session started with a single threshold estimate of “quiet threshold,” which was defined as the level supporting 50% identification of a male target presented alone at 0° azimuth. Thresholds were obtained using one-up/one-down tracking and a three-stage tracking method. Initially, the level was set at 50 dB SPL and reduced in level by 10 dB until a single error was made, at which point the level was increased in 5dB steps until the sentence was correctly identified, after which six reversals were measured using a 1dB step size, and the threshold was the average of these last six reversal values. No listeners were tested for whom this initial threshold exceeded 45 dB SPL.

Responses were obtained using a touch screen monitor located on a stand within arm's reach of the listener seated in the middle of the anechoic chamber. The monitor was directly in front of the listener but below the plane of the loudspeakers. The listener initiated each adaptive track and indicated the color and number keywords associated with the target callsign “Charlie” by selecting a virtual button with the appropriate colored number from among a grid of the 32 possible color/number combinations. The names of the colors were written to the side of the rows of colored buttons to ensure that colorblind participants could use the interface. Feedback was given after each presentation in the form of “Correct” or “Incorrect.” Approximately one second of silence followed the response being registered, prior to the next stimulus presentation.

All participants completed the testing in at least three sessions of no more than 2 h each. Participants were encouraged to take breaks and initiate each run only when they felt ready, which resulted in some variability in the total test time, especially between the oldest and youngest listeners. All procedures were approved by the Portland VA Medical Center Institutional Review Board and all participants gave written consent and were monetarily compensated for their time.

### Participants

Thirty-four listeners participated, varying in age from 25 to 74 years (mean of 50.3 years, standard deviation of 15.6 years). All had hearing thresholds of 50 dB HL or better at octave frequencies of 2 kHz and below in both ears (mean values varied from 11.3 to 18.6 dB HL, with standard deviations of no more than 12.5 dB). At 4 and 8 kHz, greater losses were present (thresholds of up to 95 dB HL in a few listeners), but all listeners had pure-tone averages (0.5, 1, 2, and 4 kHz) that were all below 32 dB HL, and all had fairly symmetrical hearing at 2 kHz and below (no differences exceeding 10 dB at more than one frequency, and no differences exceeding 20 dB at any frequency).

### Results

Quiet thresholds were found to vary between 16 and 41.5 dB SPL and were not significantly correlated with age (*R*^2^ = 0.05, *p* > 0.05), although quiet speech thresholds were significantly correlated with the average pure-tone thresholds at the two ears. Correlations were stronger for the average of three low- to mid-frequency thresholds (5, 1, 2 kHz: *R*^2^ = 0.62, *p* < 0.0001) than for three mid- to high-frequency thresholds (1, 2, 4 kHz: *R*^2^ = 0.52, *p* < 0.0001).

**Figure 1:**
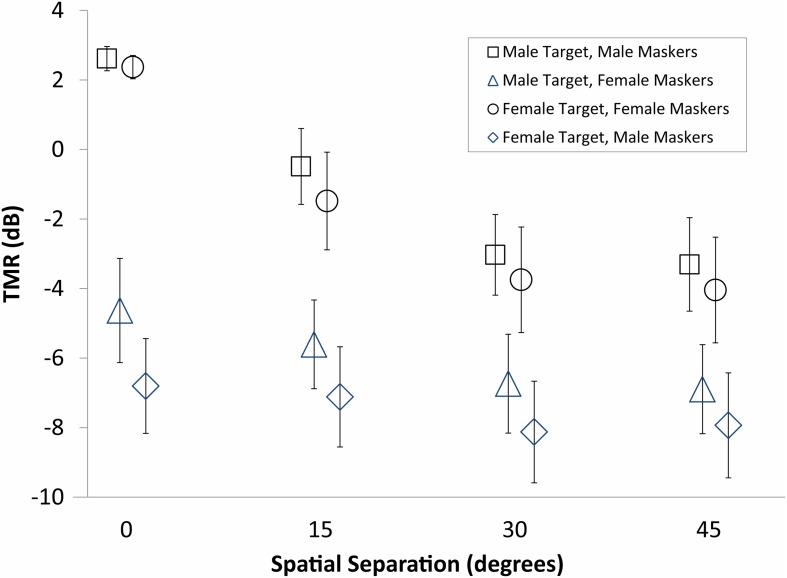
**Average thresholds for the subjects in Experiment One**. Target-to-masker ratio (TMR) is plotted as a function of spatial separation between targets and maskers for male and female targets identified in the presence of male and female maskers. Spatial separation of 0° corresponds to colocated targets and maskers presented from the same loudspeaker.

Data were analyzed with a within-subjects analysis of variance, (ANOVA; SPSS v.20) in which between-subject variables were entered as covariates. Target (male, female), masker (same gender, different gender), and spatial separation (0, 15, 30, 45°) were entered as within-subjects factors and the between-subjects factors of age and identification threshold in quiet (“quiet threshold”) were entered as covariates. Quiet threshold was chosen as the most influential measure of hearing sensitivity based on exploratory stepwise regression with a variety of potential predictors, including individual frequency thresholds and various pure tone averages. Quiet threshold was found to be significantly correlated with target-to-masker threshold in each condition (values ranging from 0.32 to 0.68, *p* < 0.05). Statistically significant effects are shown in Table 1. Mean values as a function of target, masker, and spatial separation are shown in Figure 1. As shown in Table 1, the main effects of each were statistically significant and the variance accounted for (as measured by partial eta-squared) was greater than 35%.

**Table 1:** **Within-subjects ANOVA for Experiment One**.

	**Degrees of freedom**	***F***	***p*-value**	**Partial eta squared**
**TESTS OF BETWEEN-SUBJECTS EFFECTS**				
Quiet threshold	1,31	21.336	<0.001	0.408
Age	1,31	15.062	0.001	0.327
**TESTS OF WITHIN-SUBJECTS EFFECTS**				
Masker	1,31	29.149	<0.001	0.485
Spatial separation	3,93	28.281	<0.001	0.477
Target	1,31	18.417	<0.001	0.373
Masker * Spatial separation	3,93	20.035	<0.001	0.393
Spatial separation * Quiet threshold	3,93	8.782	<0.001	0.221
Masker * Spatial separation * Age	3,93	5.166	0.002	0.143
Masker * Spatial separation * Quiet threshold	3,93	3.366	0.022	0.098

Between-subjects factors entered as covariates. Greenhouse-Geisser corrections for violations of the assumption of sphericity were conducted for the spatial separation factor (the only within-subjects factor with more than two levels), but the results were unchanged. Statistical interaction effects that failed to reach significance are not shown.

The between-subject factors of age and quiet threshold were both statistically significant and accounted for more than 32% of the variance in TMR. Interactions among the between-subject factors (age and quiet threshold) and the within-subjects factors were non-significant for all two-way combinations with the exception of spatial separation and quiet threshold. The only significant interaction of within-subject factors was the two-way interaction of masker and spatial separation. Both between-subjects factors formed three-way interactions with masker and spatial separation. None of the other three-way interactions or any four-way interactions were significant.

**Figure 2:**
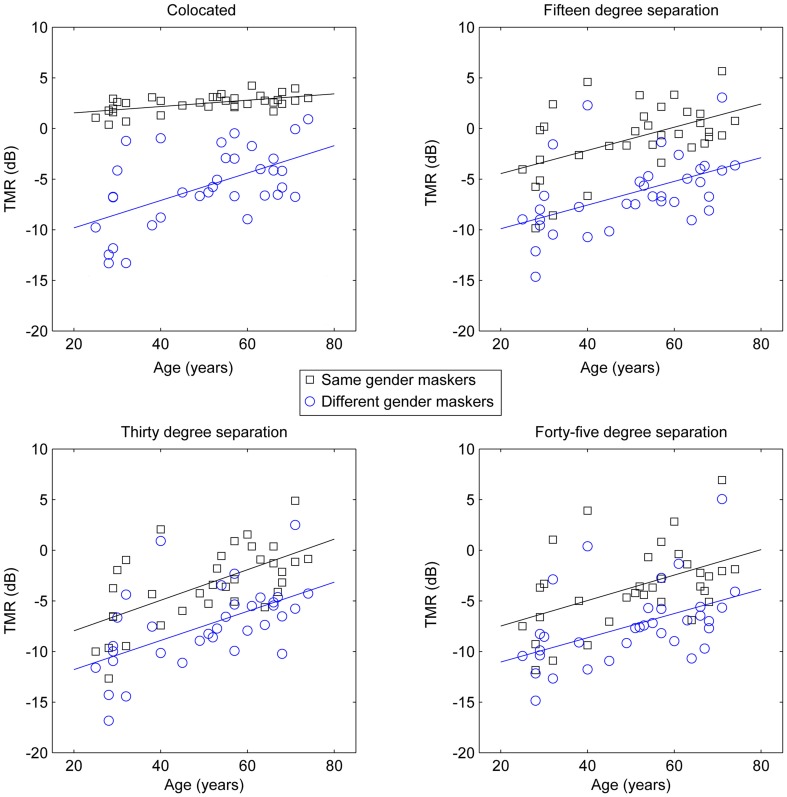
**Target-to-masker ratio (TMR) plotted as a function of age for the subjects in Experiment One**. Each panel plots same-gender maskers (averaged across target gender) as squares and different-gender maskers as circles.

**Figure 3:**
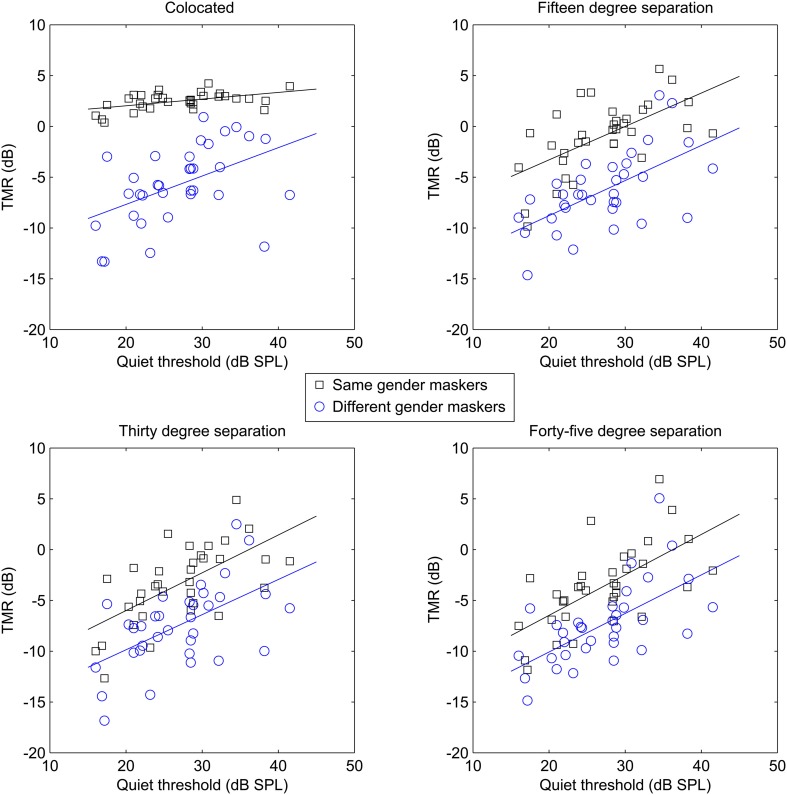
**Target-to-masker ratio (TMR) plotted as a function of quiet threshold for the subjects in Experiment One**. Each panel plots same-gender maskers (averaged across target gender) as squares and different-gender maskers as circles.

To further examine the cause of the significant interactions, *post-hoc* trend analysis was conducted. The three-way interaction of masker, spatial separation, and age was found to be based on linear effects of all three factors (*p* < 0.01, partial eta-squared = 0.23), as was the case for the three-way interaction of masker, spatial separation, and quiet threshold (*p* < 0.05, partial eta-squared = 0.18). In an attempt to illustrate the interactions among these factors, as well as the basic main effects, which are obvious across multiple combinations of factors, Figure 2 shows target-to-masker threshold as a function of age for the two masker types at each of the four spatial separations (one per panel, all panels), while the four panels of Figure 3 demonstrate the same relationships for masker, quiet threshold, and spatial separation.

### Discussion

These results strongly support the hypothesis that age and hearing loss are independently responsible for reduced spatial release from masking. Figures 2, 3 demonstrate this by showing that the patterns of results are fairly similar for age and quiet threshold, despite the low correlation the between two factors (less than 5% shared variance). In both cases, colocated TMR values are fairly similar across listeners for the conditions where target and masker are the same gender, but much greater variability is observed when either the genders are different or the spatial separation is greater than 0°. This can be seen most easily by comparing the error bars for the same gender maskers in the colocated condition in Figure 1 with the error bars for all other conditions.

Both the high variability and the relatively strong relationship with age and/or hearing loss have been observed in recent publications (Marrone et al., 2008; Strelcyk and Dau, 2009; Neher et al., 2011; Glyde et al., 2013), but in each of these previous studies the small sample size, the heterogeneity of the study sample, and/or the complexity of the statistical analyses attempted has resulted in reduced or non-existent support for the hypothesis that aging has an influence on spatial release from masking independent of hearing loss. In each case, multiple regression was conducted and, once the variance accounted for by one factor has been partialled out, no further variance could be explained by the other factor. To examine this issue, multiple stepwise linear regression was conducted on the TMR data from each condition (reported in Gallun and Diedesch, 2013). As in previous work, both factors were significant contributors for many of the conditions. Unlike in previous work, when only one factor accounted for all of the variability, that factor was age. The proportion of variance accounted for by age and hearing loss (alone or in combination) was between 22 and 54%.

## Experiment two: adaptive tracks; virtual space

To improve the methods available for predicting individual sensitivity to spatial cues, the impacts of hearing loss were further minimized by using headphone presentation, which permitted greater control over the audibility of the stimuli. A virtual spatial array (VSA) was used to test the hypothesis that spatial release testing can produce the strong effects of aging seen in Experiment One without the use of an anechoic chamber. It was also hypothesized that the impacts of even mild hearing loss would be reduced by equating audibility of the stimuli at the two ears, and that even stronger effects of aging would be revealed.

### Materials and methods

The methods were very closely matched to those of Experiment One, with a few notable exceptions. The first difference was the use of a VSA presented over insert earphones (Etymotic ER2). The VSA is a technology that depends on imposing the appropriate binaural time and level differences on a given source signal such that the resulting signals at the ears of the listener will resemble the signals that would occur if the sources had been presented from a loudspeaker at a given location (Xie, 2013). The approach used here was to use head-related impulse response (HRIR) measurements and MATLAB functions available from the Music and Audio Research Laboratory, at New York University (http://marl.smusic.nyu.edu/wordpress/projects/scanir/) and convolve each CRM wavefile to be presented with left ear and right ear HRIRs measured for a binaural manikin. The main differences between the VSA and loudspeaker presentation would have involved the spectral differences among locations and between listeners associated primarily with differences in vertical location. As none of the stimuli were simulated to be emanating from vertical locations off of the horizontal azimuth, it is unlikely that large differences in perceived location were present across listeners. Furthermore, the data from Experiment One indicate that once the spatial separation exceeds 15°, spatial separation only mildly affects performance. Consequently, even if one listener were to perceive a spatial separation of 40° and another listener 50° (which would be fairly unlikely), the impacts on performance might still be too small to detect with these methods.

The second important modification to the methods associated with Experiment One was the use of equal sensation level (SL) signals. This was achieved by first measuring the level at which each listener could just identify speech presented by the audiologist over the audiometer, transforming that value from hearing level (HL) to dB SPL by adding 22 dB, and then adding 30 dB to that level to obtain the level of the target sentence, which was always fixed during the adaptive tracking procedure. As is shown in the results section below, this approximation for the HL to SPL transform resulted in a range of values in dB SPL that were essentially identical to the range of Quiet CRM values obtained in Experiment One. The two masker sentences were again presented at levels relative to the target, so they were appropriately scaled in SL as well. No listeners were tested for whom the 30 dB SL level would have resulted in maskers that exceeded 85 dB SPL.

In addition, minor changes were made to the protocol to reduce testing time and improve the accuracy of the estimates. Based on the results of the first experiment, performance was evaluated for the colocated (0°) and 45° spatial separation conditions, and the two conditions with female targets were not included. This eliminated roughly three-quarters of the conditions that would have to be tested, thus, allowing the length of each adaptive track to be extended by two reversals (to eight rather than six) and to repeat the entire set of tracks, resulting in eight rather than four adaptive tracks contributing to the estimate for each condition.

Responses were obtained using a monitor located on a table in front of the listener. The listener initiated each adaptive track and indicated the color and number keywords associated with the target callsign “Charlie” by using a mouse to depress a virtual button using the same interface as in Experiment One. Feedback was given after each presentation and the next trial was initiated roughly one second after the response was registered. All participants completed the testing in no more than 2 h, and only occasionally was the testing divided into two sessions. All procedures were approved by the Portland VA Medical Center Institutional Review Board and all participants gave written consent and were monetarily compensated for their time.

### Participants

Fifty two listeners participated, varying in age from 19 to 76 years (mean of 45.3 years, standard deviation of 17.0 years). Nine of these individuals had participated in Experiment One and all had recently completed a similar listening experiment also using the CRM sentences with the same spatial separations as were employed in this experiment, thus, all of the listeners were very well trained on the task. All had hearing thresholds of 40 dB HL or better at octave frequencies of 4 kHz and below in both ears (mean values varied from 7 to 14 dB HL, with standard deviations of no more than 11 dB HL). At 8 kHz, greater losses were present (thresholds of up to 80 dB HL in a few listeners), however, the mean thresholds were all below 21 dB HL. All listeners had fairly symmetrical hearing at all audiometric frequencies. None had differences exceeding 10 dB HL at any one frequency or differences exceeding 5 dB HL at more than one frequency. Speech Reception Thresholds (SRTs) were tested by reducing the level of speech until listeners could only correctly report 50% of the words spoken. Thresholds obtained in this standard clinical test varied between 0 and 15 dB HL at the left ear (mean of 8.17 dB HL) and −5 and 20 dB HL (mean of 6.54 dB HL) at the right ear.

### Results

SRT values were set for each ear independently and ranged between 17 and 42 dB SPL, which is extremely similar to the range of SPL values found for the quiet speech in Experiment One (16–41.5 dB SPL). Target levels, which were set 30 dB above the SRT, varied from 47 dB SPL to 72 dB SPL, reflecting the 25 dB range of SRT values in the sample. In this case, SRTs at the left and right ears were significantly correlated with age (*R*^2^ = 0.30, *p* < 0.001 for the left; *R*^2^ = 0.10, *p* < 0.002 for the right) and were again significantly correlated with audiometric thresholds, as would be expected. All correlations were between *R*^2^ = 0.16 and 0.81, with *p* < 0.05 for each. These values do not reflect corrections for multiple comparisons, but the majority of the correlations would still have been significant.

A within-subjects ANOVA (SPSS v.20) was used, with between-subject continuous variables entered as covariates. The within-subject factors were spatial separation (0 vs. 45°) and masker (same vs. different gender). Age and SRT (averaged across ears) were between-subject factors entered as covariates. Statistical results are shown in Table 2. Mean TMR thresholds for the colocated conditions were 2.0 dB (standard deviation of 1.6 dB) for the same-gender maskers and −6.9 dB (standard deviation of 3.4 dB) for the different-gender maskers. When the same-gender maskers were spatially separated, the mean TMR was −7.8 dB (standard deviation of 3.2 dB) and when the different-gender maskers were spatially separated the mean TMR was −10.8 dB (standard deviation of 3.5 dB). These values are substantially better than what was observed in Experiment One, by as much as 4.5 dB in the case of the same-gender maskers at 45° separation. Possible explanations for this difference are discussed below, but the most likely is the use of the equal SL target, which may have improved performance for many of the hearing-impaired participants.

**Table 2:** **Within-subjects ANOVA for Experiment Two**.

	***F*_(1, 49)_**	***p*-value**	**Partial eta squared**
**TESTS OF BETWEEN-SUBJECTS EFFECTS**			
Age	16.848	<0.001	0.256
SRT	7.37	0.009	0.131
**TESTS OF WITHIN-SUBJECTS EFFECTS**			
Spatial separation	300.015	<0.001	0.86
Masker	172.016	<0.001	0.778
Spatial separation * Masker	80.01	<0.001	0.62
Masker * SRT	10.454	0.002	0.176
Spatial separation * SRT	7.937	0.007	0.139
Spatial separation * Masker * Age	4.414	0.041	0.083
Spatial separation * Age	4.031	0.05	0.076

Between-subjects factors entered as covariates. Statistical interaction effects that failed to reach significance are not shown.

**Figure 4:**
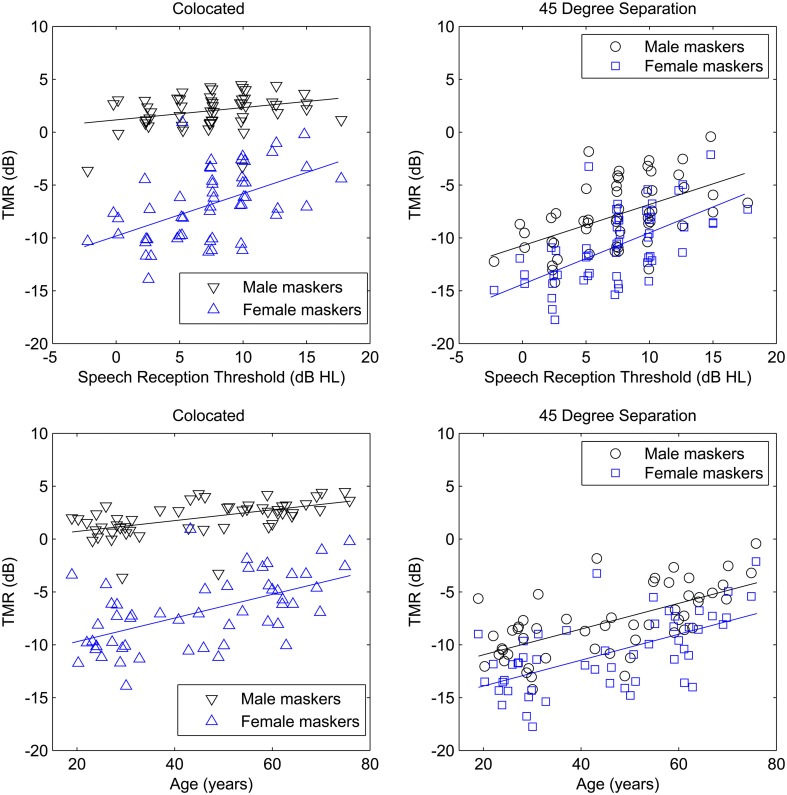
**TMR as a function of speech reception threshold (SRT; top panels) and age (bottom panels) for the participants in Experiment Two**. Thresholds for male and female maskers are plotted separately for colocated targets (left panels) and spatially separated targets (right panels). SRT values are offset slightly to improve visualization.

The main effects of spatial separation and masker type were both statistically significant and the variance explained (measured by partial eta-squared) was greater than 75%. Main effects of age and SRT were also statistically significant, although age accounted for 26% of the variance while SRT accounted for only 13%, again likely due to the use of equal SL stimuli. Figure 4 illustrates these main effects by plotting TMR for the four conditions as a function of age and SRT.

Three interactions among the within and between-subjects factors were able to account for greater than 10% of the variance: the spatial separation by masker interaction, the SRT by masker interaction, and the SRT by spatial separation interaction. Figure 4 also illustrates these interactions.

### Discussion

The results of Experiment Two also support the hypothesis that age reduces spatial release from masking independently of hearing loss. Indeed, age accounted for nearly twice the variance than did SRT, suggesting that equating the audibility of the stimuli across listeners and across ears allowed the effects of age to be even more apparent. Furthermore, these results indicate that the use of HRIRs measured with a binaural manikin allows the creation of a virtual acoustic space with sufficient veridicality to capture the same amounts of spatial release and between-subject differences in performance as were found in the anechoic chamber presentation used in Experiment One. In fact, listener performance was better in the virtual acoustic space, both in terms of the average thresholds and in terms of the best performance achieved. This is likely due to two factors: the better overall hearing of the participants (PTAs for the frequencies 0.5, 1, and 2 kHz were 15.1 dB in Experiment One and 9.4 dB in Experiment Two) and the use of an equal SL target set at 30 dB above individual SRTs for each ear. However, it should be noted that there may have been a learning factor involved, as all of the participants in Experiment Two had already spent several hours performing a slightly different version of this task (not reported here) and thus, had substantially more practice than the listeners in Experiment One. Another possible explanation is that the HRIR convolution introduced additional cues that were of benefit to the listeners, although it is more often the case that actual loudspeaker presentation results in better performance than that found with generic HRIRs.

Regardless of the reason for the improved performance associated with headphone presentation, it is clear from these data that older listeners perform less well, on average, in these tasks than do their younger counterparts, especially when the effects of hearing are sufficiently controlled through subject recruitment, sample size, and amplification of the stimuli. In addition, these data show that the VSA can support very good performance in a spatial release from masking task.

## Experiment three: progressive tracks; virtual space

The final experiment was designed to examine whether or not a rapid presentation of fixed TMRs could be used to reveal the same effects of age, independent of hearing loss, as were shown in the first two experiments with longer adaptive tracks. The hypothesis to be tested was that the effects of age and spatial separation were both so great that even a very rapid test with a minimal number of experimental trials could reveal the same pattern of performances across listeners as was found with more traditional methods. This would provide evidence both for the strength of the aging effects and for the utility of a rapid testing procedure that could be used either in combination with a larger battery of tests or could even be adapted for clinical use.

### Materials, methods, and participants

To reduce testing time, only the condition involving a male target with male maskers was examined. This was justified by the very similar results that were obtained for the two same-gender masking conditions, but removes the different-gender masking condition. This parameter (same- vs. different-gender) was not found to be as sensitive to the effects of aging and hearing loss as was the spatial separation parameter, and the interaction between gender of the masker and spatial separation was also not found to produce particularly large effects. Consequently, it was decided that comparing the colocated same-gender maskers with the spatially-separated same-gender maskers would provide the strongest test of the suitability of the progressive track for identifying differences across listeners.

The “progressive” tracking procedure involved the presentation of 20 trials, two at each of 10 TMR values, starting with 10 dB TMR and decreasing in steps of 2 dB until reaching a level of −8 dB TMR. This set of fixed TMRs would allow the stimuli to be designed in advance and presented via a fixed-track sound system such as a CD player or smart phone (provided the sound fidelity was appropriately verified). In this experiment, however, the tracks were still randomized for each participant and presented using the same computer-controlled audio system used in Experiment Two. Responses were recorded in the identical manner as well. From the perspective of the participant, the only difference was that the task started at a very easy TMR and became progressively more difficult, rather than moving back and forth between being relatively easier and more difficult across the length of the track. Using the VSA, each participant completed one progressive track with colocated maskers followed by one progressive track with maskers spatially separated from the target by plus and minus 45°.

Rather than measuring reversals, as in the adaptive procedure, performance was estimated using the much simpler metric of counting the total number of trials in which the color and number were both reported correctly. This was also used to provide a preliminary threshold TMR estimate by subtracting the number correct from 10 dB. At the extremes, this metric can be shown to be logically appropriate because if the number correct is zero, then the TMR is certainly no less than 10 dB, and if the number correct is 20, then the threshold is likely to be around −10 dB (given that the scale has an accuracy of roughly 2 dB). The appropriateness of this metric was evaluated by comparing the performance of each listener with the threshold estimate for that same listener in Experiment Two. To facilitate this comparison, the same 52 participants from Experiment Two all participated in Experiment Three.

### Results

A within -subjects ANOVA (SPSS v.20) was conducted on the estimated thresholds, with the between-subject continuous variables of age and SRT entered as covariates. The estimated threshold is based on the transformation described above, but as it is a linear subtraction, the same statistical results would be obtained from an analysis of the number of correct responses. Thresholds are reported primarily as they are more easily compared with the previous data. Statistical results are reported in Table 3.

**Table 3:** **Within-subjects ANOVA for Experiment Three**.

	***F*_(1, 49)_**	***p*-value**	**Partial eta squared**
**TESTS OF BETWEEN-SUBJECTS EFFECTS**			
Age	24.211	<0.001	0.331
SRT	0.022	0.882	0
**TESTS OF WITHIN-SUBJECTS EFFECTS**			
Spatial separation	55.91	<0.001	0.533
Spatial separation * Age	7.274	0.01	0.129
Spatial separation * SRT	0.33	0.568	0.007

Between-subjects factors entered as covariates.

As opposed to the 16 thresholds obtained in Experiment One and the four in Experiment Two, only 2 thresholds were obtained: the colocated condition (mean of 2.13 dB, sd of 2.2 dB) and the 45° separation (mean of −4.3 dB, sd of 3.8 dB). The colocated threshold is quite similar to the thresholds found in the first two experiments (2.6 and 2.0 dB), while the separated threshold is better than the −3.3 dB found in Experiment One and not as good as the −7.7 dB found in Experiment Two.

**Figure 5:**
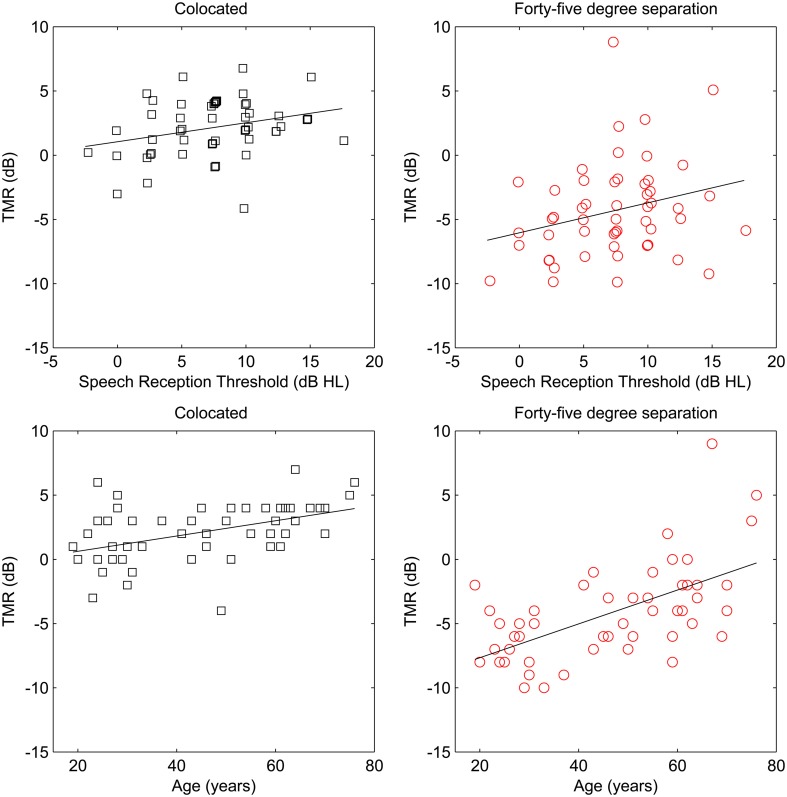
**Target-to-masker ratio (TMR) plotted as a function of speech reception threshold (SRT; top panels) and age (bottom panels) for the participants in Experiment Three**. To further reduce testing time, only the same-gender maskers were tested. Although all targets and maskers were male, colocated target, and maskers (left panels) and spatially separated target and maskers (right panels) are plotted separately to illustrated the ability of the progressive tracking procedure to capture the same range of thresholds as were found in the first two experiments. SRT values are offset slightly to improve visualization.

The main effects of spatial separation and age were both statistically significant, but the main effect of SRT was not. The interaction of age and spatial separation was significant, but the interaction of SRT and spatial separation was not. The relationships of age and SRT to thresholds in the two conditions are illustrated in the four panels of Figure 5.

### Discussion

Two aspects of the results of Experiment Three are of particular note. The first is that in this experiment the effect of age completely eclipses the effect of hearing loss as a relevant factor. Not only does SRT fail to reach statistical significance, it literally explains 0% of the variance (see Table 3). The second result of note is that the range of thresholds revealed by the progressive tracker is similar to the ranges obtained in the first two experiments. This suggests that a rapid testing approach with fixed TMR values can reveal the same differences between subjects as can the adaptive approach.

## General discussion

The results of these three experiments clearly demonstrate that the impacts of aging on spatial release can be substantial and can exist completely independently of effects of hearing loss, provided that the experiment is sufficiently powered due to a large sample size and a relatively narrow set of statistical tests. This provides one of the strongest pieces of behavioral evidence to date in support of the hypothesis, based on animal work (e.g., Walton, 2010), that age-related changes in the temporal properties of brainstem nuclei (likely along with cortical changes) can result in difficulties in auditory scene analysis independent of those associated with hearing loss. Future work using a battery of peripheral, central auditory processing, and cognitive tests will be needed to more fully distinguish these effects across a range of tasks, but the data presented here pave the way for further work on the central auditory processes associated with aging *per se*.

**Figure 6:**
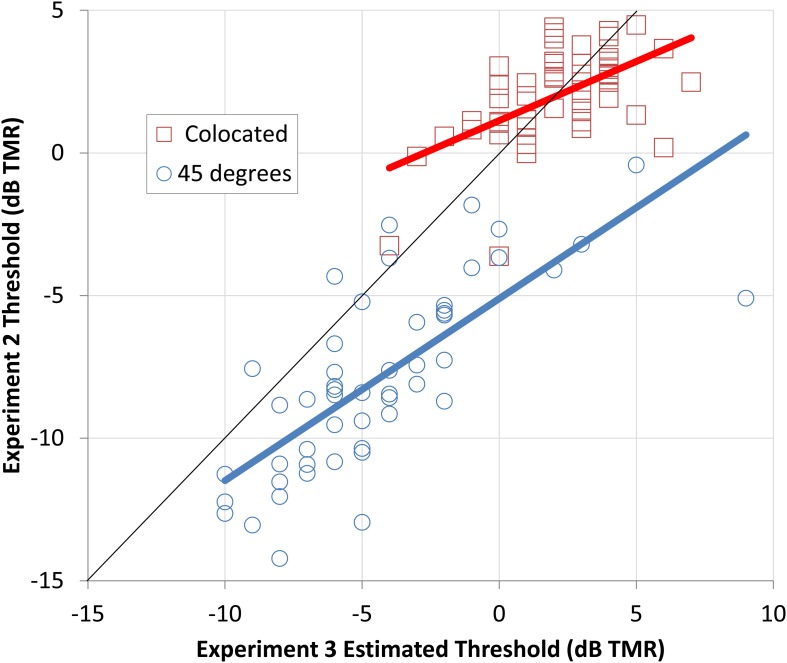
**The thresholds obtained in Experiment Two (y axis) for the male target and male maskers are plotted relative to the thresholds estimated from the progressive tracks used in Experiment Three (x axis)**. The squares indicate the thresholds for the colocated conditions and the circles indicate the spatially separated condition. Best-fitting linear predictors are shown as thick red (colocated) and blue (spatially separated) lines.

In addition, it is clear that the CRM corpus can be used to rapidly and efficiently assess the amount of spatial release a listener can achieve. By transforming the signals with HRIRs that are freely available for download, a VSA was presented over headphones and the performance of an individual listener could quickly be estimated. The relationships between performance in the conditions from Experiment Three and the same conditions as tested in Experiment Two are shown in Figure 6.

The relationship is strongest for the spatially separated conditions (*R*^2^ = 0.55), but the colocated conditions are also strongly related (*R*^2^ = 0.32). The better thresholds for the spatially separated condition in Experiment Two can be seen by the large number of points falling below the solid black line. These results indicate that the progressive tracking provides fairly accurate estimates of performance in the colocated condition, but underestimates the amount of spatial release a listener is likely to experience. Nonetheless, the progressive tracking appears to provide a rapid method of distinguishing those who are better at separating talkers in a complex auditory scene from those who are worse at this important communicative task.

## Conclusions

These data show clearly that the impacts of age on spatial release from masking can be substantial and independent of the amount of hearing loss a listener suffers. This finding supports the hypothesis, drawn from animal models (e.g., Walton, 2010), that aging results in changes in the temporal processing occurring in brainstem nuclei essential for the encoding of acoustical cues associated with spatial locations of sound sources. To identify these changes in the cortical and subcortical structures of individual patients, and improve communicative rehabilitation strategies, it is important that clinical tests of spatial hearing be developed. Such tests should be designed so that very little time is taken away from the standard clinical test battery, and should use methods that are easily understood by patient and clinician alike. It is hoped that the approach developed in our laboratory and described here can eventually be incorporated into the clinic both as a diagnostic tool and as an outcome measure to assess rehabilitative approaches such as the benefit of a new hearing aid fitting or prescribed auditory training regimen.

### Conflict of interest statement

The authors declare that the research was conducted in the absence of any commercial or financial relationships that could be construed as a potential conflict of interest.

## Footnotes

^1^Note that this is different from the signal-to-noise ratio, which is the ratio of the target to the combined level of the two maskers, which in this case would be −3 dB, due to the uncorrelated signals in the two maskers. Acoustic measurements of the combined maskers confirmed that the summed signals were, on average, 3 dB higher than either alone. Due to the multiple summation combinations possible at each ear with the various spatial configurations, it is generally preferred to refer to TMR in studies of speech on speech masking when spatial separations are employed.
