# Corrigendum: Independent impacts of age and hearing loss on spatial release in a complex auditory environment

**DOI:** 10.3389/fnins.2014.00264

**Published:** 2014-08-22

**Authors:** Frederick J. Gallun, Anna C. Diedesch, Sean D. Kampel, Kasey M. Jakien

**Affiliations:** ^1^Department of Veterans Affairs, National Center for Rehabilitative Auditory Research, Portland VA Medical CenterPortland, OR, USA; ^2^Otolaryngology / Head and Neck Surgery, Oregon Health and Science UniversityPortland, OR, USA; ^3^Hearing and Speech Sciences, Vanderbilt UniversityNashville, TN, USA

**Keywords:** spatial hearing, aging, hearing loss, virtual spatial array, sensation level

In Gallun et al. (2013), the sensation levels of the stimuli were incorrectly calculated. The calculation described on page 6 was implemented correctly, but the conversion from HL (hearing level) to SPL (sound pressure level in standardized units) was not correct for the headphones used in the experiment. Rather than adding 22 dB to the HL value, it would have been correct to add 12.5 dB to the HL value (ANSI, 2004). Consequently, the value reported in sensation level (SL), which is the level in dB relative to the speech reception level, was reported as 9.5 dB lower than is correct.

The following changes should be made to clarify this issue. Bold text indicates where changes have occurred.

1. The sentence that begins on the end of page 5 and continues onto page 6 should read: This was achieved by first measuring the level at which each listener could just identify speech presented by the audiologist over the audiometer, transforming that value from hearing level (HL) to dB SPL by adding **12.5 dB**, and then adding **39.5 dB** to that level to obtain the level of the target sentence, which was always fixed during the adaptive tracking procedure.
2. The final sentence in the first paragraph of page 6 should read: No listeners were tested for whom the **39.5 dB** SL level would have resulted in maskers that exceeded 85 dB SPL.
3. The second sentence in the Results section on the top of page 6 should read: Target levels, which were set **39.5 dB** above the SRT, varied from 47 dB SPL to 72 dB SPL, reflecting the 25 dB range of SRT values in the sample.
4. The third sentence in the second column of page 7 should read: This is likely due to two factors: the better overall hear- ing of the participants (PTAs for the frequencies 0.5, 1, and 2 kHz were 15.1 dB in Experiment One and 9.4 dB in Experiment Two) and the use of an equal SL target set at **39.5 dB** above individual SRTs for each ear.

## Conflict of interest statement

The authors declare that the research was conducted in the absence of any commercial or financial relationships that could be construed as a potential conflict of interest.
